# Twenty-year survival of gastric cancer with peritoneal metastases using long-term normothermic intraperitoneal 5-fluorouracil and systemic mitomycin C: A case report

**DOI:** 10.1016/j.ijscr.2019.07.002

**Published:** 2019-07-12

**Authors:** Paul H. Sugarbaker

**Affiliations:** Center for Gastrointestinal Malignancies, MedStar Washington Hospital Center, 106 Irving St., NW, Suite 3900, Washington, DC, 20010, USA

**Keywords:** 5-fluorouracil, Mitomycin C, Intraperitoneal chemotherapy, HIPEC, NIPEC, Case report

## Abstract

**Background:**

Primary gastric cancer that presents with peritoneal metastases has few options for successful treatment.

**Methods:**

A simplified treatment plan started with total gastrectomy and hyperthermic intraperitoneal chemotherapy. After placement of an intraperitoneal port combined intraperitoneal and systemic chemotherapy was given for six months.

**Results:**

The treatments were tolerated well and associated with an acceptable quality of life and no adverse events. The patient is a 20-year survivor of her disease.

**Conclusions:**

Gastric cancer with peritoneal metastases was successfully treated with a combination of gastrectomy plus intraperitoneal and systemic chemotherapy. Further studies using this simplified approach may be indicated.

## Highlights

• Primary gastric cancer presenting with peritoneal metastases has limited successful treatment options.
• Treatments utilize aggressive systemic cancer chemotherapy with profound negative impact on quality of life.
• A simplified management plan using gastrectomy, HIPEC, and long-term intraperitoneal and systemic chemotherapy was used.
• Her quality of life throughout treatment was excellent and resulted in a 20-year survivor of these treatments.

## 1 Introduction

Gastric cancer with peritoneal metastases diagnosed at onset of the malignancy is a formidable challenge for successful treatment. Survival following this diagnosis is usually less than one year even if the primary cancer can be resected [1]. Approximately 10% of gastric cancers will present with peritoneal metastases and an additional 16% with positive peritoneal cytology [2,3]. Three different strategies are currently in use at different institutions. The American strategy uses systemic chemotherapy in an attempt to achieve significant regression of the cancer [4]. If by radiologic study the disease seems to be under control partial or total gastrectomy may be performed especially if the patient is symptomatic and unable to take adequate nutrition orally.

A second approach is currently pursued in Japan having been initiated by Yonemura and coworkers and now pursued by Ishigami and his multi-institutional team [5,6]. Laparoscopy is used to assess the extent of disease and then place an intraperitoneal port. Repeated doses of intraperitoneal chemotherapy using a taxane (either docetaxel or paclitaxel) is used for approximately 3 months before repeat peritoneal cytology and laparoscopy are performed. If the peritoneal surfaces are cleared of peritoneal metastases, gastrectomy with peritonectomy will be performed.

The third option is gastrectomy as the initial treatment with hyperthermic intraperitoneal chemotherapy (HIPEC) [7]. This strategy has been shown to improve survival in a randomized controlled trial by Yang et al. in Beijing [8]. The HIPEC treatment was with cisplatin and docetaxel. Median survival was increased from 6.5 to 11.0 months (p = 0.046).

In the patient reported here the diagnosis of diffuse peritoneal metastases from linitis plastica was made by gastroscopy and laparoscopy. Total gastrectomy followed soon thereafter with HIPEC cisplatin and doxorubicin. Soon after recovery long-term intraperitoneal chemotherapy was initiated. The patient is a 20-year survivor and maintains an excellent quality of life.

Data on this patient was prospectively recorded and then retrospectively reviewed at an academic institution. This research work has been reported in line with the SCARE criteria [9]. This study was registered as a case report on the www.researchregistry.com website with UIN 4846.

## 2 Case presentation

In August 1999, a 68 year old, otherwise healthy Caucasian woman, complained of post-prandial pain. An upper gastrointestinal Barium study showed an obvious deformity. Gastroscopy could not confirm a stomach cancer. Laparoscopy showed diffuse peritoneal metastases. Physical examination showed no Virchow’s node or epigastric mass with deep palpation. On rectal examination there was no mass in the cul de sac (rectal shelf). The patient’s performance status was 0. CA125 was 113 with CEA and CA 19-9 within normal range.

In February 2000, the patient underwent total gastrectomy with greater and lesser omentectomy and splenectomy. The dissection was a D1 lymphadenectomy [10]. Intraoperatively, the patient received cisplatin at 85 mg and doxorubicin at 26 mg at 42 °C for 90 min as HIPEC. Following HIPEC reconstruction was by end-to-side esophagojejunostomy with Roux-en-Y jejunojejunostomy. The patient recovered without adverse events.

Pathology report showed the laparoscopy port site infiltrated by poorly differentiated adenocarcinoma. The gastric cancer was in the antrum of the stomach and diffusely infiltrating the stomach wall. There was lymphatic permeation and neural invasion by tumor cells. There were tumor cells on the serosal surface at both proximal and distal margins of resection. Four of five lymph nodes showed microscopic invasion. The greater omentum showed foci of cancer cells with a desmoplastic stroma. The surface of the appendix, gallbladder and spleen showed metastatic adenocarcinoma. A splenic lymph node and left gastric lymph node separately submitted showed microscopic metastatic disease demonstrated by immunohistochemical stain for cytokeratin. Stage was T3N2M1.

In April 2000, six weeks post-gastrectomy in the operating room under general anesthesia, the patient had an intraperitoneal port inserted without incident. Her CA 125 tumor marker had decreased to 5.6. Two days after post-placement chemotherapy with intraperitoneal 5-fluorouracil (900 mg/day for 5 consecutive days) and systemic mitomycin C at 14 mg on day 3 of the cycle was administered. This was repeated for a total of 6 cycles with 3 weeks between treatments [11].

In April 2019, the patient has no evidence of disease at age 88.

## 3 Discussion

Cancer chemotherapy for gastric cancer with peritoneal metastases is rarely effective long term. This may result from the rapid progression of disease before adequate doses of chemotherapy can be delivered. A more successful use of chemotherapy in gastric cancer is prevention of disease in patients with serosal invasion by the primary cancer [12,13]. This successful prevention strategy uses perioperative intraperitoneal chemotherapy with gastrectomy to eradicate the most common site for surgical treatment failure of gastric cancer which is the peritoneal metastases.

The strategy used in this patient may be the simplest and most patient friendly. After confirming the diagnosis of peritoneal metastases by laparoscopy, a resection of the laparoscopy port site and D1 gastrectomy is performed. The HIPEC is performed open immediately following resection with heat and chemotherapy to prevent cancer cells from implanting in suture lines or the abdominal incision. The intestinal reconstruction follows the HIPEC [14]. As soon as possible (6 weeks in our patient) an intraperitoneal port is placed surgically and combined intraperitoneal 5-fluorouracil and systemic mitomycin C begun [15]. The rapid transition of intraoperative chemotherapy (HIPEC) to normothermic intraperitoneal chemotherapy (NIPEC) may be a crucial component of this treatment strategy. A non-radical gastrectomy (D1) is thought to allow the fastest return of intestinal function in Caucasian gastric cancer patients [10].

Many systemic chemotherapy regimens with high toxicity have been advocated for gastric cancer patients [16]. Many of these are restricted to patients shown not to have peritoneal metastases. This is because responses of peritoneal metastases to systemic chemotherapy are less than responses at other sites [17]. This observation provides the rationale for direct intraperitoneal chemotherapy administration into the peritoneal space in patients with established peritoneal metastases.

There has existed for decades a competition between all systemic chemotherapy for gastric cancer treatments versus a regional (intraperitoneal) chemotherapy approach. No doubt that all systemic chemotherapy is simpler for the patient and more convenient for the oncologist. The requirement for long-term peritoneal access free of adverse events has never been achieved. Safe and effective repeated access to the peritoneal space remains an unsolved problem in gastric cancer treatment plans [15,18]. Yet the data regarding the peritoneum as the most common site (49%) for gastric cancer progression remains [13]. This high incidence of local-regional treatment failure persists despite preoperative cancer chemotherapy [19].

The single administration of chemotherapy in the operating room as HIPEC is unlikely to maintain a durable disease remission. The HIPEC is considered a necessary component of prevention or treatment of peritoneal metastases from gastric cancer but not sufficient for prolonged benefit. The rapid initiation of combined intraperitoneal and systemic chemotherapy treatments may be crucial for a long-term remission. A phase 2 trial to test general applicability of a bidirectional strategy is indicated.

## Funding

Secretarial support and data management support funded by Foundation for Applied Research in Gastrointestinal Oncology.

## Ethical approval

Local IRB-approval for this case report was not required:

MedStar Health Institutional Review Board has determined that a case report of less than three (3) patients **does not meet the DHHS definition of research** (45 CFR 46.102(d)(pre-2018)/45 CFR 46.102(l)(1/19/2017)) **or the FDA definition of clinical investigation** (21 CFR 46.102(c)) and therefore are not subject to IRB review requirements and **do not require IRB approval**.

This case report is of 1 patient.

## Consent

Written informed consent was obtained from the patient for publication of this case report and accompanying images. A copy of the written consent is available for review by the Editor-in-Chief of this journal on request.

## Author’s contribution

Paul H. Sugarbaker, MD: study concept or design, data collection, data analysis or interpretation, writing the paper.

## Registration of research studies

This case report is registered as a case series on the www.researchregistry.com website with UIN 4846.

## Guarantor

Paul H. Sugarbaker, MD.

## Provenance and peer review

Not commissioned, externally peer-reviewed.

## Declaration of Competing Interest

The author has no conflicts of interest to declare.
